# Transition support for patients admitted to intensive treatment for anorexia nervosa: qualitative study of patient and carer experiences of a hybrid online guided self-help intervention (ECHOMANTRA)

**DOI:** 10.1192/bjo.2023.642

**Published:** 2024-04-16

**Authors:** Danielle Clark Bryan, Katie Rowlands, Pamela Macdonald, Valentina Cardi, Suman Ambwani, Jon Arcelus, Sabine Landau, Ulrike Schmidt, Janet Treasure

**Affiliations:** Centre for Research in Eating and Weight Disorders (CREW), Institute of Psychiatry, Psychology and Neuroscience, King's College London, UK; Centre for Research in Eating and Weight Disorders (CREW), Institute of Psychiatry, Psychology and Neuroscience, King's College London, UK; and Department of General Psychology, University of Padova, Italy; DIS Study Abroad in Scandinavia, Copenhagen, Denmark; Institute of Mental Health, University of Nottingham, UK; and Bellvitge Biomedical Research Institute (IDIBELL), Hospitalet del Llobregat, Barcelona, Spain; Department of Biostatistics and Health Informatics, King's College London, UK; Centre for Research in Eating and Weight Disorders (CREW), Institute of Psychiatry, Psychology and Neuroscience, King's College London, UK; and South London and Maudsley NHS Foundation Trust, London, UK

**Keywords:** Anorexia nervosa, eating disorders, aftercare, transition, intervention

## Abstract

**Background:**

Adults with anorexia nervosa experience high levels of relapse following in-patient treatment. ECHOMANTRA is a novel online aftercare intervention for patients and carers, which provides psychoeducation and support to augment usual care.

**Aims:**

To explore patient and carer experiences of receiving the ECHOMANTRA intervention.

**Method:**

This is part of the process evaluation of the ECHOMANTRA intervention as delivered in the TRIANGLE trial (ISRCTN: 14644379). Semi-structured interviews were conducted with 20 participants randomised to the ECHOMANTRA (ten patients and ten carers). Thematic analysis was used to analyse the interview transcripts.

**Results:**

Five major themes were identified: (1) Mixed experience of the intervention; (2) tailoring the intervention to the stage of recovery; (3) involvement of carers; (4) acceptability of remote support; and (5) impact of self-monitoring and accountability.

**Conclusions:**

Participants were mostly positive about the support offered. The challenges of using remote and group support were counterbalanced with ease of access to information when needed. Components of the ECHOMANTRA intervention have the potential to improve care for people with eating disorders.

Recent reports have highlighted the need to improve care for people with eating disorders, given the high mortality rate due to physical health complications and suicidality seen in individuals with serious and/or long-standing mental illness^1,2^ and the uncertainties about treatment.^3,4^ Increased demand^5^ and resource shortages have led to a harm reduction approach, with shorter admissions, with limited weight restoration^6^ as documented in a recent systematic review of parameters of in-patient care.^7^ However this approach might allow the illness to persist, as discharge weight is a strong predictor of outcome.^8^

The transition from intensive treatment can be problematic^1^ with 20–50% of participants relapsing,^9^ mainly within the first 60 days after discharge.^10^ A UK parliamentary report relating to concerns about the management of transitions within eating disorder services ^11^ was followed by research examining the management of transitions from home into universities,^12^ and transitions from in-patient care to home^13^ with specific feedback from parents,^14^ siblings and partners.^15^ Parents explained how information sharing was often confusing and beset with logistical problems involving geographical, funding or time constraints, and described as an obstacle the ‘confidentiality’ issue after the age of 18. Parents requested that support be tailored to the family structure to consider siblings and separated/divorced parents.^14^ Siblings and partners raised their specific difficulties.^15^

The TRIANGLE trial was a pragmatic randomised control trial designed to examine the impact of adding a digital aftercare intervention (ECHOMANTRA) for patients and carers.^16^ ECHOMANTRA targeted the processes identified in the maintenance of anorexia nervosa mapped in the cognitive interpersonal model.^17^ ECHOMANTRA consists of an intervention directed at carers (‘Experienced Carers Helping Others’: ECHO) and an intervention for patients (‘iMANTRA’). ECHO has been shown to produce benefits in terms of reducing carer distress and length of treatment in preliminary studies in both adult and child and adolescent populations.^18,19^

The patient pathway ‘iMANTRA’ is a digital guided aftercare adaptation of the Maudsley Model of Anorexia Nervosa Treatment for Adults (MANTRA^20^) augmented by recovery narratives which were piloted in the ‘Self-Help Aid and Recovery Guide for Eating Disorders’ pilot trial.^21^ A proof of concept study found that the hybrid intervention ECHOMANTRA showed promise.^22^

The ECHOMANTRA materials consist of an online workbook, a library of video clips, including lived experience recovery narratives and role-plays of carer skills, and facilitated and moderated live online groups (patient-only, carer-only and joint patient–carer groups). Carers also received a copy of a more detailed carer guide, ‘Skills-based caring for a loved one with an eating disorder’.^23^ A detailed description of the intervention is shown in the supplementary material with Supplementary 1 available at https://doi.org/10.1192/bjo.2023.642.

The aim of this study was to explore and describe participants’ and carers’ experiences of the ECHOMANTRA intervention and to elicit feedback about potential improvements.

## Method

### Ethics statement

The authors assert that all procedures contributing to this work comply with the ethical standards of the relevant national and institutional committees on human experimentation and with the Helsinki Declaration of 1975 as revised in 2008. All procedures involving human subjects/patients were approved by Camberwell St Giles – Research Ethics Committee [16/LO/1377]. All patient and carer participants provided written informed consent. This paper is reported in line with the Standards for Reporting Qualitative Research.^24^

### Participants

Participants were recruited from the TRIANGLE trial cohort (patients aged 16 or above, admitted for specialist intensive eating disorder treatment and their carers; full details on eligibility criteria, setting and recruitment procedure are available in the protocol paper^16^). Participants who were in the ECHOMANTRA arm of the trial for a minimum of 6 months (*n* = 135) at the time of recruitment (April 2020) were eligible for inclusion in this study. The sample was non-purposive, in that participants were not selected based upon their level of engagement with the intervention. Participants were invited by email and were scheduled to participate in an interview on a first come, first served basis.

### Measures and materials

#### Interview schedule

A semi-structured interview schedule was developed by the research team, including two researchers with lived experience of the illness and one researcher with lived experience of caring and expertise in qualitative research. The interview involved questions for both patients and carers about the specific intervention components, including the online groups, self-help materials (workbook, videos), research components (questionnaires, feedback) and overall experience. For the full interview schedules, see the supplementary material, Supplementary 2.

#### Interview procedure

The interviews lasting 45–90 min via Skype teleconference were conducted by a researcher who was independent from the participant-facing TRIANGLE team. All interviews were undertaken during the first COVID-19 lockdown period between April and October 2020.

### Data analyses

The data were analysed using thematic analysis with inductive coding.^25^ A critical realist approach was used which focuses on a scientific method of data analysis, whilst accepting that the understanding of the researchers is inevitably constructed by their personal experiences and perspectives.^26^ Further details can be found in the supplementary material, Supplementary 3.

## Results

### Participant characteristics

Twenty participants were recruited, with 10 patients and 10 carers, of whom four were ‘dyads’ (patients and carers from the same family). Participants were aged 18–35 (mean = 24.10, s.d. = 4.86), of whom all (100%) were female and of White British ethnicity, with low body mass index (mean = 15.89, s.d. *=* 1.16) and a duration of illness ranging from 2 to 13 years (mean = 6.60 years, s.d. = 4.03). Carers were aged 48 to 63 (mean = 53.20, s.d. = 5.12). Nine out of ten (90%) carers were female, and all (100%) were of White British ethnicity. Nine carers were parents of the individuals involved in the trial, and one was a partner. The demographics and clinical features were reflective of the full sample described in the main trial paper (Cardi et al, in preparation). Participants were interviewed on average 9.5 months after they joined the trial (in days, mean = 293, s.d. = 64) from 14 in-patient treatment (80%) or intensive day care (20%) services located across England. Eight were NHS and six non-NHS centres.

### Summary of patients’ and carers’ experiences of the ECHOMANTRA intervention

Five superordinate themes and 19 subthemes were identified, as summarised in Table 1. (To preserve anonymity, personal references have been removed from quotations). Although most of the feedback about the intervention was positive, a major theme was that a more personalised approach might be helpful, matching the features of the participants, such as their readiness to change and the duration and severity of the illness. The inclusion of written and video material from people with lived experience was particularly noted to be helpful, as was the educational content for carers. Nevertheless, some carers and patients described demoralisation and exhaustion due aspects of the illness such as striving for perfection. For the most part, there was recognition that ongoing support, possibly including a mixture of digital and tailored materials, could be used to sustain the progress made from in-/day patient care.

**Table 1:** Themes and sub-themes identified from interviews with a sample of patient and carer participants in the TRIANGLE study

Theme number	Theme	Subtheme	
		Positive subtheme	Negative subtheme
1	Mixed experience of the intervention	Impact of identifying and connecting with others Utility of videos and transcripts	Triggering and draining
		Promoting communication Contributing to treatment development	
2	Tailoring the intervention to the stage of recovery	Matching resource to stage of change	Unhelpful for pre-contemplation stage
3	Involvement of carers	Carers increasing skillset New insights and perspectives especially from joint patient/carer work	Carer exhaustion Challenges with joint patient–carer participation
4	Acceptability of remote support	Accessible and flexible resources Facilitator guidance Online anonymity	Technical challenges and rigidity of groups Online anonymity
5	Impact of self-monitoring and accountability	Improved self-awareness	Practical and emotional effort Pressure for perfection

#### Theme 1: mixed experience of the intervention

Although many participants appreciated the benefits of the intervention, a few reported some aspects as triggering, tiring and draining.

##### Impact of identifying and connecting with others

All participants who attended the online groups reported valuing ‘a community of people around you that were facing similar things’ (Patient 9). Feeling supported by peers (emotionally and practically) was cited as an integral benefit of participation. Sharing experiences created a sense of community, and repeated attendance of group members (via username recognition) over time increased familiarity despite participant anonymity. This sense of connection was present even if people did not join the groups in real time but only read the transcripts:

‘*I feel support from the other carers, you know, if you're having a bad day and you just really struggling, you get a lot of support when everyone else must be feeling the same. It's really good to know that you're not alone’*. (Carer 6)

Several patients and carers described finding value in contributing to the peer community by offering emotional support to others and using their own experiences to offer practical advice:

‘*Sometimes it is great to hear other people, and when you're in a stronger mind frame sometimes you can offer support or advice or your own reflections’*. (Patient 1)

Participants who had not actively participated in online groups also expressed feeling a supportive peer community through reading transcripts of past groups or reading comments on the open forums:

‘*The fact that people were supporting each other … and when someone asked a question that maybe I would have asked, even though I wasn't there to ask it, it was quite nice to see other people's answers to it’*. (Patient 5)

##### Utility of videos and transcripts

This sub-theme reflects participants’ experience (particularly that of patients) connecting with the lived experience of others:

‘*It's great to have a video saying “It's fine if you feel like this at this stage right now and that doesn't mean that you're not actually recovering” or ‘It doesn't mean that you're completely relapsing if this happens … it's that kind of information and normalising all of these thoughts’*. (Patient 1)

Carers valued the videos as an introduction to the online groups:

*‘Having those little videos just gives you a little snapshot and they're the right length to just get you thinking’*. (Carer 4)

##### Promoting communication

Some patients and carers reported improved communication at home, which they primarily attributed to the online groups and improved communication skills:

‘*Because we both do it … take part in some of the TRIANGLE project, I think it's brought us closer and has just made us much more open and patient in the way that we communicate and approach each other’*. (Carer 1)

‘*It just gives us a framework for talking ourselves about, about issues’*. (Carer 10)

##### Contributing to treatment development

A major incentive for engaging was to support the research, hoping that documenting and sharing their experiences would help others:

‘*There needs to be a dramatic difference in the care that you get in transition and in hospital and when I'm out of hospital, so I think it's just a good thing to have research for, and my answers will hopefully have some impact somewhere along the line’*. (Patient 5)

Another incentive was a sense of directly helping other participants within the online group discussions:

‘*I feel like I'm actually kind of doing something positive to help towards [recovery from] eating disorders, in general, so I think for me it's a very personal thing’*. (Carer 6)

##### Triggering and draining

Personal challenges included feeling triggered during the online groups:

‘*It was a bit difficult when say someone had gone backwards … because you try and block that out and try and focus on yourself and your own recovery*’. (Patient 7)

Some competitiveness was perceived within the patient-only groups:

‘*The patient-only ones being a little bit competitive and a bit triggering … but to be honest, I've been in a lot of like face-to-face support groups as well and it's always the same’*. (Patient 3)

#### Theme 2: tailoring the intervention to the stage of recovery

Participants highlighted the importance of accessing the materials through different stages of change.

##### Matching resource to stage of change

All participants commented on the value of having the intervention resources available when needed, such as during gaps in treatment or when participants were no longer able to access services, e.g. during transition, or as a supplement to previous information already acquired:

‘*When I first came out of the hospital, I was using quite a lot of the resources. There was quite a lot going on, so I think it's probably at that time that I used the resources more, when I was sort of struggling a bit more to find my feet again*’. (Patient 9)

Many carers expressed the wish to have received similar resources earlier in the course of the illness. Most still found the resources useful; however, others felt it was too late to use them:

‘*If there had been more of this sort of thing early on in her recovery then I really do think that that would have been helpful … I don't necessarily think there's a lot that my daughter can get from this anymore because I think it's all too entrenched’*. (Carer 7)

##### Empowering

Over half of the participants identified progress in their recovery journeys linked to engagement with the intervention:

‘*[Engaging with the intervention] kinda made you feel a bit empowered; it gave you “Ooh there's something I can do to help myself in this situation” … It gives you a bit of hope and inspiration’*. (Patient 9)

Carers also reported on improvements in their loved one's motivation in recovery, e.g. ‘She is becoming more responsible for her wellbeing.*’* (Carer 8). In turn, carers spoke about becoming less reliant on support as their loved one's recovery improved:

‘*More recently I've not joined them (online groups), because we've moved on … you know things have got so much better’*. (Carer 2)

##### Unhelpful for pre-contemplation stage of change

All patients recalled challenges engaging with the intervention when experiencing ambivalence about continued recovery:

‘*It's hard for someone with an eating disorder too, when you don't have the motivation to use [the tools], especially if it's self-help … it's good when you're at the stage of motivation to change’*. (Patient 10)

Some patients and carers expressed indifference towards their participation, particularly in cases where the illness was more entrenched:

*‘I think sometimes if you've been ill for a very long time – I've had this disorder for 13 years and I think it can feel a bit disheartening sometimes because you feel that there's a message that's maybe not for someone like you’*. (Patient 4)

Carers also expressed hopelessness:

‘*They're fine, useful but at the end of the day, there's nothing really made much difference’. (*Carer 7)

Other carers acknowledged continued ambivalence:

‘*My daughter's not at the stage where she wants to contribute on the forum, so it's quite interesting to see that she's not quite there yet’*. (Carer 6)

#### Theme 3: involvement of carers

Many carers reported gaining new skills, although others expressed that they were too exhausted to participate. The experience of joint patient/carer groups was mostly positive with some initial anxiety.

##### Carers increasing skillset

Carers’ determination, unconditional love and motivation were notable in supporting their loved one by adopting some of the taught skills, building support networks for themselves and offering support to others. Many carers referred to the online groups as a helpful resource for developing skills and confidence:

‘*I would use the skills and the things that I had learned to help me … I always challenged myself to take away one or two things that you would use*’. (Carer 2)

Furthermore, carers gained confidence from drawing on others for support and creating boundaries for themselves and in supporting their loved ones:

‘*I think it's actually given me the strength to battle some of the battles that we need to [ … ] or going back and having a look at the resources and thinking “Ok yeah now is not the time” and saying things in different ways so actually I feel it's been quite beneficial’*. (Carer 6)

A few patients noticed that their carers were benefitting by using new skills :

‘*They've [online groups] been helpful for my dad … because it just makes him aware …  what to say and what not to say, just how to be around the situation’*. (Patient 8)

##### New insights and perspectives especially from joint patient/carer work

The online groups (particularly the joint patient/carer groups) produced a greater understanding of different perspectives:

‘*I particularly found it brilliant that some of the participants were sufferers, eating disorder sufferers and I, and young girls like my daughter so it helped me see their point of view’*. (Carer 2)

Patients described these experiences as beneficial because ‘it actually did make a big difference to how [my carer] was with me’ (Patient 3):

‘*I think that's quite helpful, even for me hearing the experience of other people's carers and parents, that's quite motivating’*. (Patient 5)

Participants found hearing the shared experiences of others outside their own family led to more open communication within their own relationships:

‘*[Other patients] might be saying something that our loved one feels they can't say … and if you hear how somebody else is struggling then maybe in a conversation in weeks to come, you can kind of ask questions that perhaps you hadn't thought about’*. (Carer 8)

##### Carer exhaustion

Exhaustion, lack of support and challenges with service provision were among key problems cited by both patients and supporters:

‘*I was just exhausted … I wanted to get more involved but part of me that knew emotionally and physically I couldn't do it’*. (Carer 3)

‘*My daughter's health would dip again and then along with that so would my motivation … it's the times that you really need the most support that you, you know, that you feel less likely to seek that support’*. (Carer 7)

##### Challenges with joint carer-patient participation

One of the concerns for carers in joint carer patient groups was accidentally speaking out of line:

‘*I'm worried about what to say on the ones where the patients are there as well … I don't want to say anything to upset anyone’*. (Carer 9)

Others found listening to the stories of others emotionally challenging:

‘*It's much, much harder for me to listen to the patients than the carers because sometimes their circumstances are very difficult, and for people who are not at that stage it's hard to hear what some patients are going through*’. (Carer 5)

#### Theme 4: acceptability of remote support

Mixed personal preferences were evident in terms of online platforms versus face-to-face support.

##### Accessible and flexible resources

Many participants appreciated these resources being contained in one space as well as the organised structure of the resources:

‘*I think it was quite easy because obviously it takes you right from the very beginning to the very end, so it was quite easy to sort of pick up at the stage you were at’*. (Patient 5)

Others appreciated the flexibility to dip in and out of the resources and the 24/7 accessibility from various locations:

‘*I think it's easily accessible like from home, you don't have to go out anywhere but it's just there’*. (Patient 7)

‘*Because it's a text feed I used to join them if I was [a passenger] in the car’*. (Carer 2)

##### Technical challenges and rigidity of groups

Most participants reported challenges with ‘glitchy*’* technology interfering with their use of the resources during their participation, whether due to problems with the website, personal computer issues or internet connection. Others preferred to avoid the online groups, either due to screen fatigue or preferring face-to-face support. Several participants had problems with timing constraints:

‘*The problem with [the groups] they were at certain times when my family would be eating, so it made participation a bit difficult’*. (Patient 6)

##### Facilitator guidance

The role of the facilitators and moderators of the groups was appreciated, particularly referencing their practical role in managing the group structure, as well as providing a welcoming, safe space:

‘*I thought they [the facilitators] were really good, they put the direction of what the group needed to go in. There was a start and an end, and I feel that they made people feel like it was a safe place, and everyone was included’*. (Patient 7)

Moderation was also welcomed by participants due to the sensitive nature of some discussions:

‘*Oh, the moderator is very good, you need to do that because this is such a desperate time for people that they can get a bit lost in their sadness and their difficulty’*. (Carer 2)

##### Online anonymity

The matter of anonymity within the online groups was strongly debated. Some participants preferred the privacy of the anonymous online groups:

‘*[Anonymity] that's what I thought was quite good about that you didn't have to give a name and a face to it. It was just like “This is how I feel” and you weren't judged for that’*. (Patient 7)

Others, in contrast, felt that the anonymity reduced their inclination to participate in a group with strangers:

‘*I find it a bit difficult sometimes to trust or engage with something that's faceless’*. (Patient 1)

#### Theme 5: impact of self-monitoring and accountability

The narratives in this theme refer specifically to participants’ experience of completing the regular self-evaluation forms within the trial protocol.

##### Improved self-awareness

Most participants reported that the self-monitoring increased their awareness about their state of well-being and sense of progress in recovery which could be both confronting and constructive:

‘*It actually made me realise how tough I've been finding it. You don't often take time out to think about where your emotions are at … I think the longer you don't acknowledge the difficulties you're facing, the harder it is to be able to support the person you support’*. (Carer 4)

Several patients referred to completing self-report questionnaires as an opportunity to remain aware of their progress after discharge:

‘*I think … being forced to check in with myself each month while I was in hospital and then during the transition out – it sort of reminded me like how far I'd come’*. (Patient 5)

Others reported being able to pay more attention to helpful strategies that supported continued recovery:

‘*I notice little habits are different as well. … So, I've really persevered and stopped … myself from going back to certain habits*’. (Patient 8)

##### Practical and emotional effort

Many participants highlighted the challenges in making time for activities that involved self-reflection over a long period:

‘*Because it's quite a long-time frame I think people maybe do it initially and then lose momentum especially if their loved one has improved or got better or doesn't have the problem anymore’*. (Carer 9)

##### Pressure for perfection

Some participants commented that they avoided engaging with the resources due to shame about progress in recovery:

‘*Sometimes if you know something has been particularly bad, you might not want to touch on something so negative … so you do try and hide how you're coping because you feel a bit of a failure if you're struggling’*. (Carer 6)

And some patients acknowledged feeling guilty about lack of progress to report to the study team:

‘*Sometimes I really do feel that I'm having a moment where I haven't made enough progress, I haven't done what I should have done or there's no movement … I think it is a personal pressure’*. (Patient 1)

## Discussion

The feedback from both patients and carers was similar and was for the most part positive. The involvement of a carer in aftercare was welcomed. In particular, the joint patient/carer groups were noted to be both novel and illuminating, providing an opportunity to step back and observe and reflect on interactions between other patient–carer dyads. Carers appreciated learning more about the illness and being taught psychological skills such as matching communication style to stages of change and how to improve emotional regulation. Many noted with regret that the information was perhaps too little and too late. Suggestions for improvements included better signposting of resources to address the diverse needs of different caregiving roles: siblings, male carers, and partners, and of those at different stages of illness and with comorbidities (e.g. autism spectrum disorder). The burden of caregiving and screen fatigue were noted as obstacles to engagement, although some appreciated the flexibility and the convenience of digital resources.

### Strengths and limitations

Unfortunately, an important limitation is that the sample interviewed were all White. There is also limited diversity in the sample as a whole, with 94% being White.

The subgroup of participants recruited for this study was those who had been in the study for a minimum of six months and who had volunteered and were chosen on a first come, first accepted basis. This may have led to positive bias.

Also, the trial design may also have limited the representativeness of the participants. For example, the requirement to include a carer may have excluded some participants and the inclusion of only one carer (mainly mothers) may have biased the feedback from the family. Another limitation of the study was that for pragmatic and economic reasons, the intervention was delivered independently from the clinical teams as a form of aftercare augmentation. This precluded having an integrated and tailored format which was a preferred option suggested by many participants.

### Future clinical implications

This feedback had several implications for the design of aftercare intervention. The reach of interventions which include lived experience and cover the needs of diverse carers (parents, siblings and partners) at all stages of the illness, with all forms of comorbidity, could be increased by incorporating some of the digital approaches provided by ECHOMANTRA. The use of virtual support routinely offered post-COVID-19 in most eating disorder services could overcome some geographical and other barriers hindering the development of bridging interventions across different components of the service. A pilot study of an integrated cognitive behavioural therapy approach in Oxford has shown potential,^6^ and an integrated approach using MANTRA as an aftercare intervention delivered by video conference (SUSTAIN trial) is currently in progress in Germany.^27^

Both of these approaches also include monitoring of progress which was regarded by the majority of both patients and carers as a helpful component of aftercare. This also aligns with recommendations from a Dutch aftercare intervention.^28^ However, some caution is warranted as some participants noted that it triggered perfectionist traits. The positive feedback particularly about the joint (patient and carer) groups resonates with the literature pertaining to multifamily groups in the child and adolescent setting ^29^ and that endorsing their value in adult settings also.^30,31^ Feedback also adds to the literature highlighting the important support offered by people with lived experience.^32,33^ Integrating virtual forms of delivery would increase the reach of these interventions and facilitate the integration of personalised models of care across service pathways.^34^

## Supporting information

Clark Bryan et al. supplementary material Clark Bryan et al. supplementary material

## Data Availability

The data that support the findings of this study are available on request from the corresponding author K.R. upon request.
