# Supplementary material for: Transition support for patients admitted to intensive treatment for anorexia nervosa: qualitative study of patient and carer experiences of a hybrid online guided self-help intervention (ECHOMANTRA)
Source: BJPsych Open. 2024 Apr 16;10(3):e81. doi: 10.1192/bjo.2023.642 (PMC11060069; doi:10.1192/bjo.2023.642)
Supplement: Clark Bryan et al. supplementary material [file S2056472423006427sup001.docx]

**Transition support for patients admitted to intensive treatment for anorexia nervosa: A qualitative study of patient and carer experiences of a hybrid online guided self-help intervention (ECHOMANTRA)**

**Supplementary materials**

**SUPPLEMENT 1.**

**SUPPLEMENTARY TABLE 1.** **Template for Intervention Description and Replication (TIDieR) Description and content of ECHO and RecoveryMANTRA intervention (i.e., ECHOMANTRA) for adults with anorexia nervosa and their carers**

| Study protocol | Transition Care in Anorexia Nervosa Through Guidance Online from Peer and Carer Expertise (TRIANGLE; 1) |
| --- | --- |
| Item 1- Brief name of the intervention | ECHOMANTRA: hybrid carer (Experienced Carers Helping Others; ECHO) and patient  (iMANTRA) Maudsley Model of Anorexia Treatment for Adults (MANTRA). |
| Item 2- Rationale or goals | ECHOMANTRA is underpinned by the cognitive interpersonal maintenance model for anorexia nervosa (2-4). The target is to improve interpersonal relationships by focusing on building collaboration between both patient and carer. Carers are taught communication skills and strategies for behaviour change (e.g., motivational interviewing, emotion focused therapy). Patients target processes that maintain the disorder such as the cognitive, emotional and biological factors. Recovery tips from people with lived experience build self-compassion and a new identity. |
| Item 3- What materials were used | There are separate patient and carer workbooks, a video library compiled of short, relevant video clips (n=134) and access to secure guided online chat groups (patient-only, carer-only, and joint patient-carer groups) aimed to promote skill development, emotion regulation, social connections and a recovery identity.* |
|  | The TRIANGLE trial was a pragmatic randomised control trial designed to examine the impact of adding a digital aftercare intervention (ECHOMANTRA) for patient and carers (5). Interventions for two targets (patients and carers) were developed from the cognitive interpersonal model (4). The Intervention for carers, “Experienced Carers Helping Others” (ECHO) had showed benefit in both adult and child and adolescent populations in terms of carer distress and length of treatment in preliminary studies (6, 7). The second patient pathway, “iMANTRA” was a digital, guided, aftercare adaptation of the Maudsley Model of Anorexia Nervosa Treatment for Adults (MANTRA) (8) augmented by recovery narratives which were piloted in the “Self-Help Aid and Recovery Guide for Eating Disorders” proof of concept study (9). A proof of concept study found that the hybrid intervention ECHOMANTRA with a component for both patients and carers showed promise (10). |
| Item 4- What procedures, activities or processes were used | Following randomisation, patient and carer participants allocated to the ECHOMANTRA intervention were given access to the resources on the trial website. Patients and carers were invited to attend eight discussion groups for patients or carer (as relevant) and the additional series of 4 joint patient-carer groups. Each group lasted 60 minutes, used a recovery-oriented approach with a specific theme, and integrated a discussion of at least one resource (typically a video) acting to prime group discussion. Each group was facilitated and moderated by a trained research assistant (moderators could also be psychology graduate volunteers).  The role of the facilitator is to encourage a group discussion around the information and exercises proposed in the package of self-help materials (workbook, video-clips), and keeping group discussion solution-focussed and recovery-oriented. To begin the group, the facilitator welcome the participants, introducing themselves and the moderator and what role each of them will play during the group. The facilitator also supports participants with technical issues (i.e. checking participants can see new messages, referring them to the ‘refresh’ button) and reminding everyone to respect the group boundaries. The facilitator summarises the content of the opening video and asks participants open questions. Throughout the discussion, the facilitator regularly summarises the contributions of the group and asking appropriate follow-up questions to promote further discussion.  The moderator’s role is to safeguard the group by checking that messages posted by participants adhere to the boundaries set out by the facilitator. In cases where messages adhere, the moderator will approve the messages and they will then be visible to the group. In cases where the content of a message posted is inappropriate (i.e. does not respect the group boundaries), the moderator can either 1) reject the message entirely or 2) modify the message (i.e. removing inappropriate content or personally identifiable information). In both cases, the moderator privately contacts the participant to inform them of the reason for disallowing/modifying their message and to remind them of the group boundaries. |

*Note.* *For logistical reasons we found in the first 6 months of the trial it was not possible to arrange the individual patient/carer joint family skype meetings. After discussion and feedback with the participants, the trial steering committee and co-applicants we opted to replace this meeting with a fixed bi-weekly invitation to attend a meeting in which both patients and carers were invited to attend a multi-family group. During the study we made several minor protocol changes.

**SUPPLEMENT 2.** **Interview Schedule**

Interviewer introduction and purpose of interview – to explore the experience of the patient with regards to participation in the TRIANGLE project:

“We are interested in your point of view. Please tell us openly your ideas and experience of the intervention. We are interested in whether you found the materials helpful or otherwise. We would like your honest opinion, both positive and negative aspects. If, for example, you found them unhelpful or irrelevant please say so, as we want to work on finding the best possible interventions to help you. You are the expert. We want to hear your point of view.”

Online Groups

1. Have you booked to attend the online support groups? If so, did you participate or observe? How did you find them? Prompt: What specific aspects could you relate to?
2. To what extent did you relate to other group members? Prompt: Did you feel you were there for the same reasons, same goals?
3. What were your experiences in participating in the joint patient and carer groups (in comparison to the patient-only/carer-only groups? Prompt: How did you relate to the carers/patients? How did they relate to you as someone with an eating disorder?
4. To what extent did you relate to the group facilitator? Prompt: What was their role in making you feel at ease, included, excluded?
5. To what extent were the groups recovery oriented? Prompt: Can you explain further – in what ways?
6. What impact did the comments made by others have on you in terms of motivation? Prompt: Change or challenge the ED or no effect/impact?
7. How did you find incorporating videos into the start of the group?
8. How did you feel at the end of every online session? Prompt: What aspect of the online group did you attribute this to?
9. What are some of the pros and cons of online groups? Prompt: Anonymity, facial cues, voice intonation

Materials

1. What were your thoughts on the materials provided (workbook, videos)? Prompt: How easy did you find to navigate through them?
2. What were your views on content? Prompt: Structure, placing. How did they resonate with your own personal needs?
3. In your opinion, did the components follow a logical pattern? Prompt: How easy was it for you to navigate through the workbooks?
4. To what extent was the content applicable to your own personal challenges? Prompt: What proportion did you find relevant to your own situation?

Questionnaires

1. How did you find the questionnaires and feedback? Prompt: What personally motivated you to complete the questionnaires? How did you feel after completing the questions?
2. As you know the questionnaires provides the necessary data to evaluate whether the intervention is a cost-effective service of value for the NHS. What might you think puts people off doing the questionnaires? Prompt: Are there any practical or personal obstacles that make them more difficult to complete?
3. What do you think might motivate other participants to complete the questionnaires? Prompt: Any other suggestions?

Overall experiential perspective

1. Since you joined the project, what changes, if any, have you notice to your own wellbeing?
2. Was the intervention useful at any time in your/your loved one’s recovery path? Prompt: Has your use of the resources increased or decreased? can you describe to us what aspects were useful at what times?
3. To what extent do you feel the TRIANGLE project has impacted upon any changes, on you or your carer(s)/patient? Prompt: How have these changes impacted on you? What specific aspects of the project do you feel this has been attributed to?
4. What has been your overall experience of participating in this research project? Prompt: both positives and negative aspects. What, if anything, did you get out of it?

Thank you for participating in the TRIANGLE project. This has been invaluable to us and to research into EDs, in general. Thank you also for your time today/this evening.

**SUPPLEMENT 3. SUPPLEMENTARY DATA ANALYSES**

All interviews were completed, transcribed and coded before the main trial outcome data analyses were conducted. All interviews were audio-recorded using Skype for Windows Desktop and were transcribed verbatim. All personally identifying information was removed. Two researchers read and re-read the transcripts independently in order to become familiar with the data. Next, ‘utterances’ were given initial codes, which were reviewed and refined. Using these codes, themes were identified and organised into an initial thematic framework using the software NVivo 12 (11). Six meetings were held during the coding process, in which the researchers discussed emerging patterns in the data, altered descriptive labels to better reflect the subject matter, or deleted labels if deemed irrelevant to the research question.  This continued until general agreement had been reached between the researchers that data saturation had been reached during the coding process. The thematic framework was shared and discussed with the wider research team, at which point the final superordinate themes and sub-themes were determined as coherently representing the complexity of the data.

**REFERENCES**

1. Cardi V, Ambwani S, Robinson E, Albano G, MacDonald P, Aya V, et al. Transition Care in Anorexia Nervosa Through Guidance Online from Peer and Carer Expertise (TRIANGLE): Study Protocol for a Randomised Controlled Trial. Eur Eat Disord Rev. 2017;25(6):512-23.

2. Schmidt U, Treasure J. Anorexia nervosa: valued and visible. A cognitive-interpersonal maintenance model and its implications for research and practice. Br J Clin Psychol. 2006;45(Pt 3):343-66.

3. Treasure J, Schmidt U. The cognitive-interpersonal maintenance model of anorexia nervosa revisited: a summary of the evidence for cognitive, socio-emotional and interpersonal predisposing and perpetuating factors. J Eat Disord. 2013;1:13.

4. Treasure J, Willmott D, Ambwani S, Cardi V, Clark Bryan D, Rowlands K, et al. Cognitive interpersonal model for anorexia nervosa revisited: The perpetuating factors that contribute to the development of the severe and enduring illness. Journal of clinical medicine. 2020;9(3):630.

5. Cardi V, Ambwani S, Robinson E, Albano G, MacDonald P, Aya V, et al. Transition care in anorexia nervosa through guidance online from peer and carer expertise (TRIANGLE): Study protocol for a randomised controlled trial. European Eating Disorders Review. 2017;25(6):512-23.

6. Hibbs R, Magill N, Goddard E, Rhind C, Raenker S, Macdonald P, et al. Clinical effectiveness of a skills training intervention for caregivers in improving patient and caregiver health following in-patient treatment for severe anorexia nervosa: pragmatic randomised controlled trial. BJPsych Open. 2015;1(1):56-66.

7. Magill N, Rhind C, Hibbs R, Goddard E, Macdonald P, Arcelus J, et al. Two‐year follow‐up of a pragmatic randomised controlled trial examining the effect of adding a carer's skill training intervention in inpatients with anorexia nervosa. European Eating Disorders Review. 2016;24(2):122-30.

8. Schmidt U, Sharpe H, Bartholdy S, Bonin E-M, Davies H, Easter A, et al. Preventing deterioration and relapse in severe anorexia nervosa: Randomised controlled feasibility trial of an e-mail-guided manual-based self-care programme based on the Maudsley Model of Anorexia Nervosa Treatment for Adults (work package 5). Treatment of anorexia nervosa: A multimethod investigation translating experimental neuroscience into clinical practice: NIHR Journals Library; 2017.

9. Cardi V, Albano G, Ambwani S, Cao L, Crosby RD, Macdonald P, et al. A randomised clinical trial to evaluate the acceptability and efficacy of an early phase, online, guided augmentation of outpatient care for adults with anorexia nervosa. Psychological Medicine. 2020;50(15):2610-21.

10. Adamson J, Cardi V, Kan C, Harrison A, Macdonald P, Treasure J. Evaluation of a novel transition support intervention in an adult eating disorders service: ECHOMANTRA. International Review of Psychiatry. 2019;31(4):382-90.

11. QSR International Pty Ltd. NVivo (released in March 2020). 2020.
